# Digitalization of Religion in Russia: Adjusting Preaching to New Formats, Channels and Platforms

**DOI:** 10.1007/978-3-030-42855-6_11

**Published:** 2020-11-25

**Authors:** Victor Khroul

**Affiliations:** 1 University of Helsinki, Helsinki, Finland; 2 Maastricht University, Maastricht, The Netherlands; 3 Higher School of Economics (HSE University), Saint Petersburg, Russia; Lomonosov Moscow State University, Moscow, Russia

**Keywords:** Digital religion, Mediatization, Religious institutions, Religious practices, Russia

## Abstract

Examining the “digital” as a challenge to one of the most traditional spheres of private and public life of Russians, the chapter is focused on institutional aspects of the religion digitalization in the theoretical frame of mediatization. Normatively, digitalization as such does not contradict the dogmatic teaching of any traditional for Russia religion, in Christianity, Judaism, Islam and Buddhism theologically it is being considered as a neutral process with good or bad consequences depending on human will. Therefore, functionally digital technologies are seen by religious institutions as a shaping force, one more facility (channel, tool, space, network) for effective preaching while the core of religious practices still remains based on non-mediated interpersonal communication.

## Introduction

Facing religious life and religious practices that are traditionally conservative or even archaic, the “digital” has not yet transformed the field of religion in Russia as radically and visibly as some other areas, such as business, media, education, or culture. Nevertheless, the analysis of the digital in the religious sphere does not fit into simple statements, such as that religion is ancient, traditional and therefore—“natural,” while media are modern, upgrading and therefore—“artificial”; it is far more complex (Lundby 2014).

Helland (2000) has made an important and heuristically promising distinction between “online religion” and “religion online”: religion online means the adoption of digital formats for conveying traditional religious information (dogmatic texts, worships, preaching, institutional information of all kinds), whereas online religion engages users in spiritual activity via the Internet, and this activity may be not in line with traditional religious practices and sometimes is in open opposition to them. This distinction, when applied to Russian religious life, gives a picture that is overwhelmingly dominated—quantitatively and qualitatively—by religion online, i.e. traditional discourse “repacked” into digital form and distributed through digital channels; online religion is marginal and almost invisible. The Russian Orthodox Church (ROC) more and more effectively uses digital technologies, but still utilizes the Old Slavonic language during liturgies. Muslim and Jewish communities use smartphone apps to calculate the correct time for prayers but pray in Arabic or Hebrew as in ages before. The inner, sacral religious space remains untouched by the “digital.”

Normatively, digitalization as such does not contradict the dogmatic of any traditional religion. In Christianity, Judaism, Islam and Buddhism, it is theologically considered to be a neutral process with good or bad consequences depending on human will. Therefore, functionally digital technologies are seen by religious communities first of all as one more facility (channel, tool, space, network) for effective preaching, or *Propaganda Fidei* (the Propagation of the Faith) (Campbell 2005).

This chapter consists of three basic units. The first discusses religious organizations in Russia. The second analyzes religious digital practices, while the third section examines challenges for digitalization in religious sphere. Starting from a short description of the Russian religious landscape, we analyze normative and practical aspects of digitalization in the context of religion and then examine problematic areas of this process in Russia—the digital remapping of sacred and profane, the marginalization of religious minorities, forms of anti-digital resistance and extremism in the digital space.

## Russian Religious Landscape

The Constitution of the Russian Federation is considered by experts to be liberal and democratic. It provides equal rights: “The state shall guarantee the equality of rights and liberties regardless of sex, race, nationality, language, origin, property or employment status, residence, attitude to religion, convictions, membership of public associations or any other circumstance. Any restrictions of the rights of citizens on social, racial, national, linguistic or religious grounds shall be forbidden”; and also the freedom of religion “Everyone shall be guaranteed the right to freedom of conscience, to freedom of religious worship, including the right to profess, individually or jointly with others, any religion, or to profess no religion, to freely choose, possess and disseminate religious or other beliefs, and to act in conformity with them” (Constitution of the Russian Federation 1991).

The Government generally respects these rights in practice; however, in some cases authorities impose restrictions on certain (religious) groups.

The Russian law on religion (1997) recognized for all citizens the right to freedom of conscience and faith. It underlined the spiritual contribution of Orthodox Christianity to the history of Russia, and respect to Christianity, Islam, Buddhism and Judaism as so-called traditional religions.

When it comes to determining the numbers of followers of these religions, different approaches often give contradictory results. Moreover, the most natural approach, which is based on self-identification data, works well in most Western countries but fails in Russia. In practice, only a minority of citizens actively participate in any religion. Many who identify themselves as members of a religious group participate in religious life rarely or not at all. There is no single set of reliable statistics about the religiosity of the Russian population.

According to the Pew Research Center, 71% of Russians are Orthodox Christians, 15% are not religious, 10% are Muslim, 2% are Christians of other denominations, and 1% belonged to other religions (Religious Belief 2017). But those who claim themselves to be Orthodox Christians, do not fit any traditional criteria of religiosity, such as church attendance and familiarity with basic dogmas of their faith. Radically different results are obtained by estimating the number of practicing adepts. For example, even though up to 70–80% of the Russian population identify themselves as Russian Orthodox, less than 10% of them attend church services more than once a month and only 2–4% are considered to be integrated into church life. Moreover, the coverage in mainstream media strengthens the ethnic background of the religious identity. According to the Levada-Center, a correlation between “I am Russian” and “I am an Orthodox believer” has become stronger over the last two decades (Obŝestvennoe mnenie 2013, 118). Russian sociologist D. Furman suggested that the increase in ideological uncertainty and eclecticism, with beliefs in reincarnation and astrology, ufology, energy vampires, witches, shamans and so on, demonstrates that atheism still dominates in Russia (Furman and Kaariajnen 2006).

The Russian government evidently favors “traditional” religions, and most of all the ROC with budget financing of constructing and restoring church buildings and educational and social projects, which faces critique in the public sphere. For example, human rights activists quote the Russian Constitution and insist that the ROC and other religious organization should be separate from the state. Non-traditional religions, on the other hand, are marginalized, suppressed and even persecuted as sects (for example, Jehovah’s Witnesses).

According to the SOVA Center for Information and Analysis, the trend of increasingly restrictive policies toward Protestants and new religious movements, especially Jehovah’s Witnesses intensified in 2019:

Persecution of Jehovah’s Witnesses has become more large-scale and severe. Criminal prosecution for continuing the activities of an extremist organization, de facto for continuing the profession of religion, has already affected more than 300 people. 18 of them were sentenced, half of them to prison time, including three who received six years in penal colony. This is the first time since the Jehovah’s Witnesses organization was banned that its believers were tortured during criminal investigations. Numerous rough searches and arrests and confiscation of community property continued. (Sibireva 2020)

Experts do not expect any liberalization in government policy as the year 2020 started off with new imprisonment sentences and instances of Muslim communities that suffer as a result of the enforcement of so-called anti-extremism legislation. In addition, religious groups continue to face problems in the construction of new and continued use of existing buildings, risk criminal prosecution based on the restrictions on missionary activities and are confronted with discrimination.

## Digitalization and Religion: Normative Aspects

The impact of digitalization on religious organizations and practices in Russia is best understood in the framework of mediatization. The notion of mediatization has been applied to religion by Danish scholar Stig Hjarvard (2008). He suggested that in the digital era religion can no longer be studied separately from the media, because (a) media are for most people the primary source of their religious knowledge and religious imagination; (b) some social functions of religion are now primarily the functions of media; and (c) religious institutions use media logic and media framing for their actions (Hjarvard 2008).

There are three main ways of mediatization of religions:

- Media allow, enable and assist the self-presentation of religions, observe their activities in the public interest by maintaining religious formats (broadcasting services, funerals, weddings, etc.)
- Media cover religious life (news reports, feature stories, etc.) and may have a critical approach towards some social activities or religious institutions.
- Media outlets may use religion for their own aims: selectively importing well-known religious symbols into entertainment, keeping out sacral meanings and secularizing the essence of religion. This process is out of the control of religious authorities and therefore causes many complaints and conflicts (Thomas 2015).

The first way of mediatization mentioned above is more or less self-evident and depends on the goodwill of media institutions and on audience demand. In most cases it keeps the religious format “untouched” and the media are used more as a channel of transmission rather than actively interacting with the subject. The second and the third ways presume a more active role of journalists covering religion. The process becomes more important and at the same time more problematic. Conflict and scandals are rooted in misunderstanding or in poor reporting on religious issues.

The historical analysis of religious media in Russia explicitly shows two stages: (a) a rapid development of all religious media (1990–1997) and (b) their stratification after the division of religions in 1997 into so-called traditional (Orthodox, Muslim, Jewish and Buddhist) and non-traditional (Catholic, Protestant, Hindu, new religious movements and others). Orthodox media are supported by the state, on national and regional levels. For example, Orthodox TV channel “Spas” is included into a number of federal channels transmitted all over Russia. Some of “non-traditional” religious media decided to choose the strategy of “self-silencing.”

The situation in Russian news media and public sphere regarding religious issues differs from the situation in traditional Western democracies. The differences are rooted in the understanding of press and religious freedoms. To illustrate: while up to a million French people gathered to express their solidarity with the Charlie Hebdo journalists who were killed in Paris in January 2015 by terrorists who claimed to be Muslims, a few days later 1 million Russian citizens—mostly Muslims and Orthodox Christians—came together on the streets of Grozny (the capital of Chechnya) to show their support for “Islamic values.”

In the Russian context, the mediatization of religion faces (1) ignorance towards ethics and social accountability of digital media practitioners, (2) a normatively disoriented audience with a low level of media literacy and religious practice, and (3) a predominantly secular public sphere with problems in social dialogue processing.

In ethical perspective, the Congress of Russia’s Journalists adopted a Code of Professional Ethics (1994). Journalistic standards listed in the Code are similar to those adopted by journalists worldwide. However, its norms are hardly applied or respected by the majority of journalists.

TV remains the most important medium, and it does not appear that it will lose its prominence in the near future. Russia has become a “watching nation” instead of a “reading nation,” therefore for any actor seeking to have an impact on the general audience TV remains a strategic resource. Yet, contrary to European “success stories,” the history of the attempts to create Public TV in Russia and implement it into the existing media system in the last two decades has been marked by a series of failures.

The lack of journalistic self-reflection, the low level of media’s comprehension of their social mission and the ignorance concerning possible consequences sometimes led external structures (political, economic, social) to raise their warning voices. For example, the State Duma (Russian Parliament) on January 23, 2015, called upon all journalists for more accurate and professional coverage of religious life in Russia and abroad. “The State Duma calls on all media and all journalists in Russia and foreign countries in covering events of a religious nature to be guided by the principles of ‘do no harm,’ to refer to the publication of materials that may affect and offend the religious feelings of citizens with special responsibility and sensitivity,” the Duma statement says (Gosduma 2015).

The main dysfunctions in the coverage of religious life in Russia have been confirmed by different researchers (Kashinskaja et al. 2002; Khroul 2012):

- a biased approach among journalists, tolerated by their colleagues;
- a lack of education on religious issues and therefore a lack of understanding of what is really going on;
- an urgent need of specialized media focused on religious life;
- secular media’s dependence on political and influential Russian Orthodox Church elites;
- the marginalization of religious minorities in the public sphere.

From a religious perspective, the lack of knowledge about and experience of religious life among digital media practitioners gives much more space for myths and stereotypes in digital platforms. Moreover, not only the mass media but also religions themselves have to contribute to agenda setting and to elaboration of digital mediatization mechanisms in this very sensitive sphere. In addition to difficulties of translation from the archaic language of the religious ghetto into a modern one and problems with understanding the internal functionality of religious organizations, there are some social expectations religions do not meet.

At least two problematic areas in Russian society—“religious illiteracy” of journalists and “media illiteracy” among faith communities—could be optimized with the clarification of mutual expectations from the perspective of “pluralism—dialogue—consensus” logic (Habermas 1989) (see Table 11.1).

**Table 11.1:** Religions and digital media normative expectations

	Religions	Digital media
Pluralism	• Try to ensure religious values transparency, availability of texts representing them clearly; • Seek correct articulation of their faith, use adequate symbolic systems, language and cultural codes.	• Give platforms for complete spectrum of religions and normative models (with respect to minorities); • Optimize channels and information flows.
Dialogue	• Tolerate other approaches to religion with which they are not in agreement; • Use the framework of common cultural code; • Commit themselves to participate in the dialogue, send experts to be active in the public sphere.	• Organize and support the search for new subjects of the dialogue; • Mediate, moderate, create forums for discussions; • Expand—quantitatively and qualitatively—the space for dialogue in various forms of communication.
Consensus	• Are seeking the common good; • Are optimizing the “preaching,” the presentation of their vision from the perspective of consensus.	• Consider consensus to be one of the most important goals of media; • Are peacemakers during conflicts and tensions; • Develop openness and solidarity.

## Religious Responses to the Challenge of Digitalization

Digitalization of religion is even more complex in Russia because of its poly-confessional and poly-ethnic social structure. The set of values promoted by ROC is questioned by many Russians. Yet, the ROC remains one of the most highly trusted social institutions and some anti-ROC campaigns and scandals (“Pussy Riot” punk prayer in Moscow Cathedral and others) have not significantly decreased the trust in the ROC. Experts agree that, “a common trope for self-positioning of the Church is that the ROC is a ‘state-shaping’ religion, and as such it weaves its own historical narrative with the narrative of the Russian state” (Suslov et al. 2015). Researchers emphasize the political and geopolitical components of Russian Orthodoxy and the importance of the concept of “symphony”—harmonious relations of mutual support and mutual non-interference—between Church and state (Engström 2014; Papkova 2011; Simons and Westerlund 2015).

In order to make ROC more active in the digital space, Patriarch Kirill after his election and enthronization in 2009 announced the establishment of a new *Sinodal’nyj informacionnyj otdel* (Synodal Department of Information). In 2010, an Orthodox video channel on YouTube (http://www.youtube.com/user/russianchurch) was launched, and the Department of religious journalism and public relations at Russian Orthodox University was established.

Not all of more than 1000 Orthodox media outlets (most of them have digital versions) are in line with the ROC position, and some of them have a different approach in commenting on everyday life. Some non-official outlets, like the magazine *Tat’ânin Den’* and journal *Foma*—both founded in 1995—are not official and enjoy a larger degree of freedom of discussions than what is allowed at the official resources. Web portal “*Pravoslavie i mir*” (Orthodox Christianity and the World, www.pravmir.ru), launched in 2004, is currently the leading Orthodox multimedia portal publishing news and analytical reviews, comments and interviews, audio, video, info graphics. The audience of the portal is around 2.5–3 million visitors per month, or 100–120 thousand per day.

According to Anna Danilova, the Editor-in-Chief of Pravmir.ru, there are several essential negative presuppositions in Orthodox religious identity that affect the missionary work within digital media. “Still for a religious community the process of exploring new media normally is connected with at least these potential obstacles: (1) tendency of any religious institution to be conservative in everything including the media; (2) unclear impact of the new media on the psychological state, society and interpersonal relationships; (3) tendency to interpret many innovation as ‘diabolic ones’ (one of the best cases of which was shown in the fear of many people in Russia to accept personal tax identification code, even though the Church has officially stated that it had nothing to do with the number of the Antichrist),” writes the Orthodox journalist (Danilova 2011, 20).

Chief editor of the portal “Bogoslov.ru”, archpriest and theologian Pavel Velikanov, mentioned three pros for digital activity of the Church: (1) the possibility of Christian witnessing, the ability to communicate with people looking for answers to their questions in social networks; (2) the possibility of Christian charity—according to the priest, “charitable organizations are active in networks and live through networks,” and (3) the rapid dissemination of information. Contras, according to the theologian, are the reverse side of the pros: (1) it is very difficult to verify information; it often comes from not-trustworthy and strange sources; (2) discussions are conducted in a manner that is not appropriate for Christians; (3) people spend a lot of time on the social networks and come into the real world “just to eat” (Khroul 2015; quotations below see ibid.). Danilova considered as positive the fact that social networks make it possible to get out of the “ghetto” of just the Orthodox audience and to understand the agenda, to find out what people are now interested in. A negative point is the lack of information accuracy and difficulties with verification: “fakes” rapidly spread through social networks. On the negative side Danilova also mentioned the fact that social networking presumes too quick a reaction: “People react while they still do not really understand the situation, and relationships become strained,” Danilova said and called for general “Internet hygiene.”

Well-known Russian Orthodox journalist Sergej Hudiev suggested that it is difficult to divide the “plusses” and “minusses,” because most of the advantages are at the same time disadvantages. The advantage of anonymity is that many people are able to overcome the exclusion zone between them and the clergy, but the disadvantage is that the question of anonymity removes inhibitions of the people in the network: they cease to control what they say.

Russian TV commentator Elena Žosul, speaking about the advantages, noted that social networks are main sources of news; they allow to establish useful contacts and professional relationships and allow quick collective reflection about what is happening. On the negative side, she mentioned “the overflow of information and inability to concentrate on some issue, therefore long texts are so unpopular in the network.”

In order to prevent cybercrimes and the use of the digital space for pedophilia, pro-Orthodox organization “*Liga bezopasnogo interneta*” (League for a Safe Internet) was established in 2011 with support from the Ministry of Communication of the Russian Federation. “This organization set itself the task of fighting pedophilia and extremism on the internet, mostly by hands of the so called ‘cyber-warriors’ [*kiberdružinniki*], who provoke and expose pedophiles, and report about contentious websites to the law-enforcement bodies,” underlines Russian scholar Mihail Suslov (2015, 13).

The ROC has a leading position among religious communities involved in online communication; Muslim activity is not as expanded. The biggest and most influential Muslim digital resource in Russia is the Internet portal Islam.ru, whose main goal is to protect the interests of traditional Muslims, as well as popularize the works of traditional Islamic values. It launched the first daily Islamic news feed and opened 13 thematic sections along with a full-fledged English version of the site. Beside news, Islam.ru publishes analytical articles, religious texts (in particular, prayers) and provides psychological, legal and theological advisory. The resource has pages on all popular social networks through which feedback from readers is maintained. Islam.ru opened the possibility to become a member of the Muslim community virtually. “People become Muslims because of their convictions and sincere faith. On the site, they can leave their data in order to inform the world about their decision,” said the chief editor of the Islam.ru Rinat Muhamedov (Luchenko 2008). There is a button “I accept Islam” on the Islam.ru website; pressing it is equal to publicly pronouncing the formula “There is no God but Allah, and Mohammed is His prophet.” In addition to Islam.ru there are some independent Muslim socio-political channels, such as “Voice of Islam,” “Russian Islamist”, as well as educational projects.

Jewish, Catholic and Protestant digital resources are focused mostly *ad intra*, serving local communities and those who show some interest in them. Together with other non-traditional religious media and networks, they are marginal and less visible in the Russian public sphere in comparison to the dominant Orthodox and Muslim religious communities.

The only major television project for Russian Protestants is “Television of Good News,” which began as part of the global Trinity Broadcasting Network (TBN) and now is positioning itself as an independent public broadcaster. Without any doubt, this is the biggest Protestant media resource that broadcasts via satellites and cable networks. Protestant radio “Teos” lost its frequency and is now a fully Internet-based station. Nevertheless, it is developing, inviting interesting presenters, such as Orthodox journalist Sergej Hudiev and a number of others, trying to be interesting and relevant to a wide range of audiences, not only for Protestants. Newspaper “Mirt” is a serious newspaper for ministers and parishioners, publishing reflections and sermons, sometimes not understandable to non-Protestants. There are also a number of successful printed media outlets outside Moscow and Saint Petersburg: newspapers in Yaroslavl, Penza, Yoshkar-Ola, Voronezh, Vladivostok, Irkutsk, and other cities of Russia. Among the Internet portals the leading project is Protestant.ru that presents a good example of successful migration from a printed newspaper to web portal. The press secretary of the Union of Christians of Evangelical Faith (Pentecostals) in Russia Anton Kruglikov pointed out two major visible trends in Protestant media: (1) to move content from printed media to digital platforms and (2) to address the general public, not only those who already are Protestants.

Generally speaking, there are several problematic areas in religious digital media:

1. *Subordination of journalism to public relations (PR)*. Many of the employees of religious media in Russia find themselves serving the religious institutions in terms of public relations and advertising much more than following journalistic standards. Both the employers and the employees do not find such a situation strange.
2. *Out of touch with mission and target audience*. Digital religious media fall into the trap of thinking that their structure would be “media for all,” but in reality, they find themselves with an unclear mission and target audience.
3. *Populism and primitivism*. In order to be closer to common people, digital religious media sometimes pursue populism through primitivism of the message. Such a simplification creates a distorted image of the religious reality and also “corrupts” the religious view of cultural and social issues in Russia.
4. *Conflict of formats*. Digital religious media lack a language that is clear and understandable for the general public. In many cases, because of the language secular journalists have the impression that religious world is strange and hard to cover, therefore it is underexposed and finds itself *ad marginem* of the national media system.
5. *Religious media as the “ghetto”*. Religious media still do not realize the need to be part of social dialogue. Meanwhile, media and digital culture is increasingly becoming a space of public life and cognition.
6. *Lack of professionalism is not understood as a problem*. The lack or total absence of professionalism in religious media often is not considered to be something inappropriate.
7. *Religious media are still run mostly by enthusiasts*. In many cases the editorial staff’s enthusiasm does not receive any moral (and more material) support and understanding from the hierarchy of religious organizations, and that makes synergetic strategic planning and systematic work hardly possible.

So, from a religious perspective there are evident problems with news production, channeling, transmitting, broadcasting, with interaction and understanding; therefore, the voices of religious leaders are hardly heard in society (for more on digital journalism beyond religion, see Chap. 10.1007/978-3-030-42855-6_9).

## Sacred and Profane: Digital Remapping

In the Russian digital sphere, there are two major contextual challenges for Durkheim’s *sacred-profane* dichotomy (Durkheim 1915, 47): the enforced atheization during the Communist time and, after it, the religious revival in the context of secularization. Digitalization speeds up the remapping of the social space with sacred and profane markers: some profane objects and social practices have been sacralized, while some traditional religious ceremonies and sacred objects have been profanized. Digitalization can also lead to re-sacralization, to the creation of new sacred objects, new mysteries, and new explanations for events of supernatural origin.

The last two decades of the digital era have been a time of continuous sacred-profane remapping in Russia. Russian feminist punk rock group “Pussy Riot” staged a performance in Moscow’s Cathedral of Christ the Savior in February 2012, which was stopped by church security guards. Online video sharing was essential for Pussy Riot’s performance to reach an audience and create the scandal it created. Six months after, three members of Pussy Riot were convicted of hooliganism motivated by religious hatred and sentenced to two years imprisonment. Different ecclesiastics reactions followed the “punk-prayer” by Pussy Riot. Archpriest Vsevolod Chaplin appealed to “criminal sanctions for everyone, who affronts the faithful sense,” while at the same time deacon Andrei Kuraev commented on the event on his LiveJournal in the opposite way: “If I were a sacristan of the Cathedral I would feed them with pancakes, give a cup of mead to each of them and invite them to come round for a confession. And if I were an old layman, I would pinch them a bit at parting … Just to make wise” (Kuraev 2012).

The more recent debate on “Matilda,” a film directed by the Russian film-maker Aleksei Uchitel, which tells the story of a romance between the future Tsar Nicholas II, canonized by the Russian Orthodox church in 2000, and Matilda Kshesinskaya, a teenage prima ballerina at the Mariinsky theatre in St. Petersburg, is a good example of the “ sacralization” trend in the Russian public sphere and how it is supported by media. Radical Russian Orthodox movements warned that “cinemas will burn” if Matilda was screened, because the film portrays the “holy tsar” in love scenes. In response to the threats, the largest network of cinemas in Russia in September 2017 refused to screen the film because of safety reasons. Various other spontaneous, grass-roots public initiatives in Russia (e.g. icons of Stalin painted with the nimbus as a saint, protests against digitalization in order to avoid the “number of devil” appearing in the documents) are not in line either with Church teaching or with government intentions, but widely covered by media, inspiring the sacralization of, for example Stalin or Ivan IV Terrible.

Another example—heavily rooted in digital media support—is the process of “sacralization” of Epiphany bathing (ice swimming). Ice swimming has been practiced in Russia for centuries and some historians suggest that the practice was a popular pagan tradition. Every year on Epiphany (January 19 in Russia), Russian Orthodox believers are plunged into a blessed section of frozen water three times in remembrance of Jesus’ baptism in the river Jordan by John the Baptist. In 2019, almost 460 thousand people took part in the Epiphany bath in Moscow, and over 2.4 million in Russia (for comparison—in 2018: 150 thousand in Moscow and over 1.8 million in the entire country). Russian President Vladimir Putin traditionally, year-by-year, attends a religious service and also participates in Epiphany bathing. Even the US ambassador to the Russian Federation John Huntsman, a Mormon by faith, took part in Epiphany bathing in 2018 and called this ritual “the great Russian tradition.” The Moscow authorities published on the Mayor’s website the “rules of baptismal bathing,” which did not contain a word about the religious character of the act. And the mayor of the city of Yaroslavl, with the words “you are Orthodox people”, convincingly asked the officials to lead the bathing. Generally speaking, Epiphany bathing has become a huge media event covered by all the major media in Russia and abroad—covered as religious tradition, as something all Russian Orthodox Christians are called to do, as a ritual blessed by the Church.

In fact, many Russian Orthodox bishops and priest condemned this ritual and called on believers not to take part in it and invited them to attend Epiphany liturgy instead. Bishop Evtikhy of Domodedovo put forward four reasons for this: (1) ice swimming is dangerous for the health, it contradicts the Gospel and therefore it is a sin; (2) bathing is a profanation of the sacred—blessed water; (3) bathing is not traditional for the Russian Orthodox Church and (4) it strengthens not faith, but superstitions (Evtikhy (Kurochkin) 2019). Such a negative approach to Epiphany bathing was evident in previous centuries. “Bathing violates the sanctity and contradicts to the spirit of true Christianity; therefore, it cannot be tolerated and must be condemned,” wrote priest Sergij Bulgakov in the end of nineteenth century (Bulgakov 1913).

This opinion is low profiled both by media and state authorities and therefore not heard in the public sphere. Both media and politicians gain symbolic capital during Epiphany bathing ignoring the position of priests and bishops who have never been in fact proclaimed loudly “ex cathedra,” and therefore the ROC’s ecclesial approach to Epiphany bathing is not clear and understandable for the general public in Russia.

As Kseniya Luchenko mentioned, high-quality Church-related discussions are conducted not in mainstream media, but predominantly in digital social networks. “The answer to that question is closely linked to the analysis of dialogue culture in Russian society as a whole. Social institutions and mechanisms that are supposed to ensure and sustain that dialogue are overwhelmingly out of order. However, the need to discuss, share experiences and monitor publications is still there. And social networks make it possible,” the Russian scholar suggested (Luchenko 2015, 130). Almost all of the largest Orthodox websites have pages on social networks, such as VKontakte, Odnoklassniki and Facebook. On these social networks there are special pages of ecclesiastics, groups connected to parishes, with Orthodox public associations or churches.

The analysis of the self-expressions and discussions on religious topics in the digital platforms shows that young Russians, in matters of belief/disbelief, rely mainly on their own experience and the experience of other people (family and friends), and not on faith, authority or tradition, as would be expected (Khroul 2015). The most convincing is the socio-historical explanation for this phenomenon: the Russian tradition of faith that was consistently eradicated over a fairly long period of time. Minimizing appeals to faith, tradition and authority is a “birthmark” of Russian history, which can be described in terms of “post-atheism trauma.”

Paradoxically, the Internet users in their self-expression make evident their mostly positive attitudes towards God and predominantly negative attitudes towards Orthodox Christianity and Russian Orthodox Church. The social and political activity of the ROC faces more criticism than Orthodox Christianity as a religion: for example, “ROC proposal to impose a dress code for the people of Russia,” “ROC proposes to create a criminal penalty for heresy.” This suggestion may be proven not only quantitatively but also qualitatively, with the rhetoric of users’ voices: “ROC is a business project”; “ROC, in most cases do not care about people, but about the godless government,” “I love the Orthodox religion and Orthodox culture, myself, am an Orthodox man, but terribly hate ROC.” The arguments of those who are in favor of ROC and defend it are mostly rooted in ethnic and geopolitical discourse: “I am Russian and therefore I am an Orthodox. It is natural”; “ROC is an integral part of the thousand-year history of Russia, she has always supported our morals and I will always be with her, as the rest of the true believers.”

In 2012, a content analysis study of Russian digital Internet communication texts found observable “traces” of mainstream media publications (predominantly TV) against so-called non-traditional religious organizations (Khroul 2016). Consider, for example, some opinions on Jehovah Witnesses’ (JW) activities published on the website lovehate.ru: “According to news shows, journalists covered how some sect engaged in raping children”; “Recently in the news on TV it was said that a 50-year-old man, a Jehovah’s Witness, set himself on fire. He considered himself a great sinner who had allegedly had to wash away his sins. Thus, we see what this sect leads us to”; “This is a false religion, which is no good and kills a person (religiously destructive sect)”; “This is the most vile of sects, posing as Christianity. In fact, what we have is a simple case of Freemasons.” The analysis of the texts makes visible two important things: (1) behavioral attitudes of intolerance with respect to the JW, and (2) the willingness of people to take tough repressive measures against JW from the state. In sum, this “explosive mixture” is already provoking a request to the authorities, as in the case of aggravating state–religious relations or the case for a need to find another “enemy”. It can become a “trigger” for negative measures taken not only against the JW but also against other so-called non-traditional religions, who at the current juncture come across as an easy target. Indeed, JW were banned in Russia in April 2017 by the decision on the Supreme Court, and in February 2019 the Russian court for the first time found a Jehovah’s Witness, Danish national Dennis Christensen, guilty of extremism and sentenced him to 6 years behind bars (Russian Court 2019).

From a journalistic perspective, there is a visible problem of journalistic autonomy. According to recent studies, journalists in Russia do not enjoy autonomy because of their political and economic dependence. Secondly, the challenge of objectivity is apparent, which leads to a poor and stereotyped coverage of religious life in secular media. Agenda-setting process in media is not ethical-oriented: the main players are mostly focused not on the audience or on public interest, but on political subordination and commercial profit, therefore moral issues are secondary. Therefore, religious media are not able to change the content management: “infotainment” and “advertainment” oriented media decision makers do not seem to be concerned with fitting their products into even secular moral norms, so religious norms as more strict are ever more ignored.

## Challenges of Digitalization in Religious Perspective

For religions in Russia, all visible and invisible challenges and threats of digital communication—non-hierarchical structure, lack of authority, dogmatic corruption, information noise, fake news dissemination—seemed to be not so dangerous in comparison with their advantages and benefits and therefore manageable. Therefore, the concerns of Russian religious leaders with regard to digital technologies are mostly (with some rare exceptions) focused on misuses of them in particular cases (the spread of heresies, online pornography, gaming addiction, playing Pokémon Go in church, etc.).

Nevertheless, there are visible “grassroots” protests among Russian Orthodox fundamentalists against digitalization in general. According to these fundamentalists, digitalization in the context of religion is not limited to its technological side, as it is always a threat. Moreover, for some ultraconservative Russian Orthodox Christians the “digital” as such has ontologically negative connotations related to “the number of the beast” and the process of the digitalization is seen as a visible sign of the Apocalypse, the end of the world.

Therefore, digitalization was accompanied with protests against, for example, the “barcode” or “666” digits in the passport numbers of some Orthodox believers. Paradoxically, the campaign against individual tax numbers (INN) in 2000 became the first civil action of a religious nature in Russia, in which the Internet was used as a tool of influence, the main mean of information exchange. Individual tax number opponents using digital platforms and channels brought this topic onto the agenda of mainstream media and of church–state relations (Luchenko 2008). The movements against electronic control and globalization processes are widely using one of the main tools of globalization—the Internet. While widely rumored, these views still are marginal in Russian media and public sphere.

Various semi-pagan cults and self-proclaimed “prophets,” who previously were not known beyond the regions of their activity, nowadays cover the entire territory of the country, thanks to digital network channels. In 2008, the case of the so-called Penza hermits—a group of believers who reject the foundations of modern society and the state and spent more than half a year, having closed themselves in in a dugout in the Penza region, became widely known (New Cult 2008). The spread of myths about the “sanctity” of Ivan the Terrible, Grigori Rasputin, Russian Emperor Pavel I, and voices demanding their canonization by the ROC would not be so successful without digital networks. Moreover, some informal groups that hold completely different views and have completely different goals can act in the digital space on behalf of the Orthodox or Muslim, Jewish, Catholic, Protestant communities. The general shift of these movements toward greater radicalism seems to be consequent; since the center of social and political discussion in the digital world is shifting towards oppositional radical structures, it is easier to act on the Internet, exaggerating their ideology.

Yet, the biggest concern in terms of social security in the digital space is raised by radical extremist networking. After Twitter closed more than 300 thousand accounts on suspicion of spreading extremist ideology in 2015, the followers of the so-called Islamic state (IS) became more embittered on Telegram messenger (total number of users exceeded 100 million). Telegram officials informed that they suppressed activities related to extremism in public channels, but do not monitor private chats (encrypted and secret). On November 18, 2015, Telegram announced the blocking of 78 public channels, connected with the IS extremist group (banned in Russia). Fundamentalists used 12 languages for digital extremist propaganda. After that case, the FSB (*Federal’naâ služba bezopasnosti*, Federal Security Service) head Aleksandr Bortnikov considered the possibility of restricting Russians’ access to Telegram (RBC 2015), but this initiative was not implemented at that time.

At the same time religious organizations use various digital channels of mass communication with missionary goals, as well as to maintain the integrity of the religious community and its development, to ensure the necessary information exchange in modern conditions. For the ROC, one of the main functions of the Internet is an electronic document management system that allows its structures and administrative units to more effectively coordinate their activities.

Despite the use of tablets and smartphones in order to follow the worships and using digital TV for live transmissions of religious events (some of which also became media events), and despite being involved more actively in web-based content production and consumption, for many Russians the core of religious practices still remains based on interpersonal communication.

## Conclusion

In spring 2020, the reactions of Russia’s various religions communities to the coronavirus pandemic were noticeably different, once more confirming the diversity of practices sketched in this chapter. While most places of worship were closed or switched to online services, some bishops in the Russian Orthodox Church insisted they would not stop in-person services or the tradition of kissing icons. Another traditional ritual in times of emergency took place in Moscow on 3 April: Patriarch Kirill took a miraculous icon of Maria, Mother of God, and made a round trip through Moscow, praying to save the city from the coronavirus.

Digitalization had a tremendous impact on religions practices during the pandemic as believers got a chance to participate in worships digitally at a distance. For example, Catholic masses all over Russia were broadcast online. Moreover, in the opinion of the Russian Orthodox Church, even the sacrament of confession became possible online. If a person wants to confess during self-isolation because of the coronavirus, then “in exceptional circumstances they can confess by phone or Skype,” said Metropolitan Hilarion, the head of the ROC Synodal Department for External Church Relations (RIA Novosti 2020).

The use of religious apps has brought about a diverse range of religious practices (e.g. confession by smartphone) that often fall outside traditional thinking, yet the rituals performed with these apps are felt to be authentic (Scott 2016). The digital network structure also frees users from the need to integrate into strict hierarchical systems and rigorously participate in rituals—that is, from important elements of institutionalized religions. In the wake of the turn from religiosity to spirituality, user practices have become increasingly diverse, sometimes deviating from church (in the case of Christianity) doctrines. Moreover, the individualization of religious practices leads to a situation in which church authorities lose their status as the final ethical and dogmatic referee.

Opening new channels and platforms for information flows, the digital era created opportunities and challenges both for religious institutions (new formats, genres, packages for preaching and communications) and for individual religiosity (variety of information sources, shift from interpersonal to digitally mediated communication). As this chapter has shown, digital technologies as a shaping force make religious life more transparent (challenging hierarchical information filters and church secrets), more liquid (after centuries of stability), and more ambivalent and pluralistic in terms of values and practices.
